# Identifying the Thermal Barriers of Glass Aging via Isoconversional Analysis

**DOI:** 10.1021/acs.jpcb.5c07109

**Published:** 2026-01-26

**Authors:** Vasiliki Maria Stavropoulou, Federico Caporaletti, Florian Pabst, Valerio Di Lisio, Simone Napolitano, Daniele Cangialosi

**Affiliations:** † 202635 Centro de Física de Materiales (CSIC−UPV/EHU), Paseo Manuel de Lardizabal 5, 20018 San Sebastián, Spain; ‡ PMAS, Faculty of Chemistry, University of the Basque Country (UPV/EHU), Paseo Manuel Lardizábal 3, 20018 Donostia-San Sebastián, Spain; ¶ Laboratory of Polymer and Soft Matter Dynamics, Experimental Soft Matter and Thermal Physics (EST), 26659 Université Libre de Bruxelles (ULB), Brussels 1050, Belgium; § Donostia International Physics Center, Paseo Manuel de Lardizabal 4, 20018 San Sebastián, Spain

## Abstract

We employ isoconversional analysis to gain insights on aging time-dependent thermal barriers in glasses evolving toward equilibrium. This is applied to glasses of different natures, including small molecules and polymers. Our analysis indicates that as relaxation proceeds, equilibration kinetics involves increasingly larger activation barriers. The latter equals that of the α-relaxation at the final stage of aging. In contrast, the relatively low thermal barriers at the initial and intermediate stages of aging indicate that mechanisms different from the α-relaxation mediate aging in these conditions. We discuss the nature of these mechanisms in the light of the complexity of glass aging.

## Graphical abstract

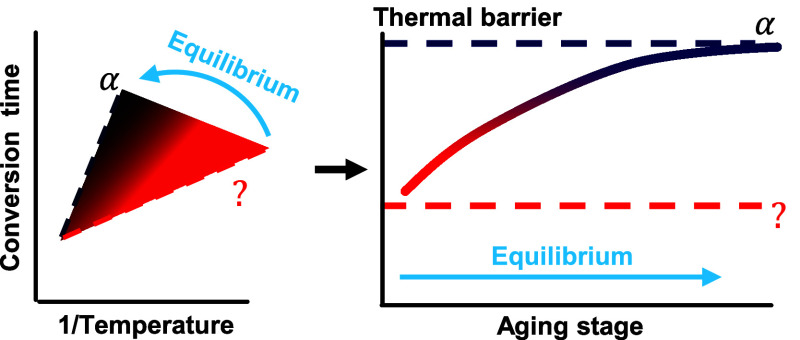

## Introduction

The transformation of a liquid into a glass is known as vitrification or glass transition.^1,2^ Over time, as a consequence of its nonequilibrium nature, the glass tends to reduce its free energy via a transformation commonly referred to as physical aging or structural recovery.^3–5^ Both vitrification and physical aging represent different aspects of the same underlying problem: the inability of the system to maintain equilibrium when molecular mobility becomes too slow on experimental time scales.

Within the common description, the microscopic mechanism ultimately responsible for both vitrification and physical aging is the main α-relaxation, a molecular process with a characteristic super-Arrhenius temperature dependence. The α-relaxation sets the structural time scale that increases steeply as the glass transition is approached and thus naturally accounts for the dramatic slowdown of dynamics observed in supercooled liquids. This idea has deep historical roots: though conceptually different,^6^ vitrification and glassy dynamics are assumed to be equivalent, with the glass transition identified as the point where the α-relaxation time reaches the experimental time scale. Accordingly, vitrification was understood as the arrest of this molecular process and physical aging as its continuation below *T*_g_.

This view holds true for specific cases of vitrification, for instance, in some polymeric systems^7–9^ and in low molecular weight glass formers,^10–12^ where cooling rate-dependent glass transition temperature, *T*_g_, and temperature-dependent α-relaxation time exhibit the same super-Arrhenius behavior. However, universality of this behavior across all materials remains debated,^13,14^ above all when confined glasses are considered.^15–19^ In the case of physical aging, the dominant role of the α-relaxation has been demonstrated in thermal protocols involving small temperature steps applied close to *T*_g_.^20–23^ These studies have shown that aging follows the same time scale as the α-relaxation, thereby reinforcing the idea that structural recovery is essentially governed by the same molecular process as vitrification. The primary implication of this finding is that because of the high activation energy associated with the α-relaxation, physical aging is expected to rapidly slow down with decreasing temperature. If aging were governed solely by the α-relaxation, one would therefore expect it to vanish not too far below *T*_g_.

However, experimental observations consistently show that physical aging occurs at detectable rate deep into the glassy state.^4^ This apparent contradiction indicates that while the α-relaxation dominates the dynamics close to *T*_g_, additional mechanisms must contribute to structural recovery at lower temperatures. Identifying the nature of these mechanisms remains one of the central open challenges in the physics of glasses.

In line with this observation, the traditional α-relaxation-based framework for physical aging has been questioned by experiments examining vitrification kinetics^13,14,16,24^ and aging behavior^23,25–28^ across broad ranges of cooling rates, aging times, and temperatures. It has been shown that the α-relaxation alone is inadequate to describe the kinetics of such phenomena, especially when it comes to physical aging substantially below *T*_g_ and over long aging time scales.^6,25^ Specifically, a wealth of experimental effort showed that long-term aging deep in the glassy state for different systems, including polymers,^25,29,30^ chalcogenide,^30,31^ and metallic glasses,^32,33^ exhibits multiple decays toward equilibrium. While the separation of multiple relaxation steps in van der Waals glasses has so far remained elusive, model-dependent analyses of aging data—based on the implementation of density scaling within the so-called single parameter aging (SPA) framework^20,34^ —have revealed the inadequacy of the α-relaxation to account for the full recovery of equilibrium deep in the glassy state.^23^ These analyses support the conclusion that more than one mechanism contributes to physical aging, although the exact number and nature of such mechanisms remain open questions.

Given these premises, a kinetic approach capable of unraveling the activation energies associated with different stages of glass equilibration is required in order to extract information on the molecular mechanisms that mediate physical aging as a function of the thermodynamic state of the glass as well as of aging time and temperature. A particularly powerful tool in this direction is the so-called isoconversional kinetics,^26,35^ which has been extensively employed in the study of a wide range of chemical reactions, including polymerization, cross-linking, thermal and thermo-oxidative degradation, and crystallization/melting processes.^36^ The extension of isoconversional methods to the study of glass transition phenomena, and in particular to physical aging,^26,27^ was pioneered by Vyazovkin et al.^37–39^

Within this framework, the kinetics of a relaxation or transformation process is analyzed by applying individual Arrhenius equations to different degrees of conversion, thereby evaluating an effective activation energy as a function of the progress of the nonequilibrium process. Instead of assuming a single constant activation barrier, the isoconversional approach maps out how the effective activation energy evolves with conversion, providing a sensitive probe of the underlying molecular mechanisms. In this way, complex temperature dependencies, which may involve a change in dominant relaxation modes or the emergence of additional mechanisms at long times or low temperatures, can be revealed through systematic variations of the effective activation energy with conversion.

An important advantage of isoconversional analysis is that it is not restricted to processes that obey simple Arrhenius kinetics. On the contrary, it is particularly suited to deal with non-Arrhenius processes, such as those typically involved in the glass transition and physical aging, where the apparent activation energy is known to evolve with temperature and observation time. Thus, isoconversional kinetics offers a rigorous and versatile framework to disentangle the different molecular mechanisms that contribute to physical aging and to quantify how their relative importance depends on thermal history and depth in the glassy state.

## Isoconversional Kinetic Analysis

Isoconversional kinetics analysis has been widely applied during the last decades for the study of various thermally stimulated processes in polymers and other molecules.^35^ It consists of a system of methods that facilitate the calculation of the kinetic triplet, i.e., the activation energy (*E*_α_), the pre-exponential factor, and transformation model, allowing at the same time making kinetic predictions and obtaining mechanistic insights. This method is particularly useful when the transformation mechanism is unknown or when complex reactions involving several steps take place. The main advantage of this methodology resides in the assessment of the activation energy at any level of conversion during the transformation. The latter can be measured with thermoanalytical techniques, the most common of which are thermogravimetric analysis (TGA), differential scanning calorimetry (DSC), and dielectric relaxation spectroscopy (DRS).^36^

As a general rule, the isoconversional method requires performing a series of experiments, where the transformation under investigation is followed at different temperatures and times. Subsequently, these kinetic data are analyzed to obtain the values of the effective activation energy as a function of conversion, *X*, which, for aging data, is normally expressed as the extent of relaxation: *R* = 1 – *X*. The activation energy at a given relaxation, *E*_*R*_, can be obtained considering the variation with temperature of the time to reach such an extent of relaxation, *t*_*R*_, in isothermal transformation kinetics data:

$$E_{R}=k_{B}{\left[\frac{\partial \ln t_{R}}{\partial T^{-1}}\right]}_{R}$$ 1

where *k*_B_ is the Boltzmann constant. Application of eq 1 to different *R*-values provides the dependence of the activation energy at different stages of the transformation under examination.

In the present work, isoconversional kinetics analysis is applied to a set of physical aging data on different glasses, including small molecules and three glassy polymers, polystyrene (PS), poly­(4-chloro styrene) (P4ClS), and poly­(4-bromo styrene) (P4BrS). In the case of small molecules and PS and P4ClS, we refer to previously reported physical aging kinetics,^23,40,41^ where the enthalpy evolution toward equilibrium was determined by fast scanning calorimetry (FSC)^42^ after rapid quenches (1000 K s^–1^) from the supercooled liquid to various aging temperatures. For P4BrS, we acquired new data using an analogous protocol (see Supporting Information for details). The extent of enthalpy change is quantified via the concept of fictive temperature, *T*_f_, introduced by Tool,^43^ defined as the intersection of the glass line, drawn from the thermodynamic state of a glass with the melt line. Hence, low *T*_f_s correspond to low enthalpy glasses and glasses completely relaxed to equilibrium exhibit *T*_f_ = *T*_a_, where *T*_a_ is the aging temperature. The extent of relaxation can be written as

$$R(t)=\frac{T_{f}(t)-T_{a}}{T_{f}(0)-T_{a}}$$ 2

where *T*_f_(*t*) and *T*_f_(0) are the time-dependent fictive temperature and the one at the beginning of the aging process, which in the case of the considered set of aging data is the *T*_f_ after cooling at 1000 K s^–1^.

## Results and Discussion

Figures 1–3, panel (a), show the aging time-dependent evolution of the *R*(*t*) function at different aging temperatures for *o*-terphenyl (OTP),^44^ P4BrS, and PS.^40^ Analogous plots, reported in the Supporting Information, are shown for all other investigated glasses, including o-cresolphthalein dimethyl ether (KDE), phenolphthalein dimethyl ether (PDE), 1,1-bis (4-methoxyphenyl)­cyclohexane (BMMPC), 1,1-di­(*p*-methoxyphenyl)­cyclohexane (BMPC), and P4ClS. As can be observed, the aging time evolution of *R*(*t*) exhibits the common patterns typical of glass aging. These are the shifts of *R*(*t*) to longer aging times with decreasing temperature and the typical sigmoidal shape. Nevertheless, the aging time evolution of *R*(*t*) appears to be more or less stretched, depending on the material. Specifically, PS appears to exhibit a more stretched aging evolution with respect to its bromo-substituted homologue, P4BrS, hinting at the influence of chemical structure on aging behavior. KDE exhibits larger time scale span at two consecutive temperatures than PDE, likely as a result of the larger fragility of the former.

**Figure 1:**
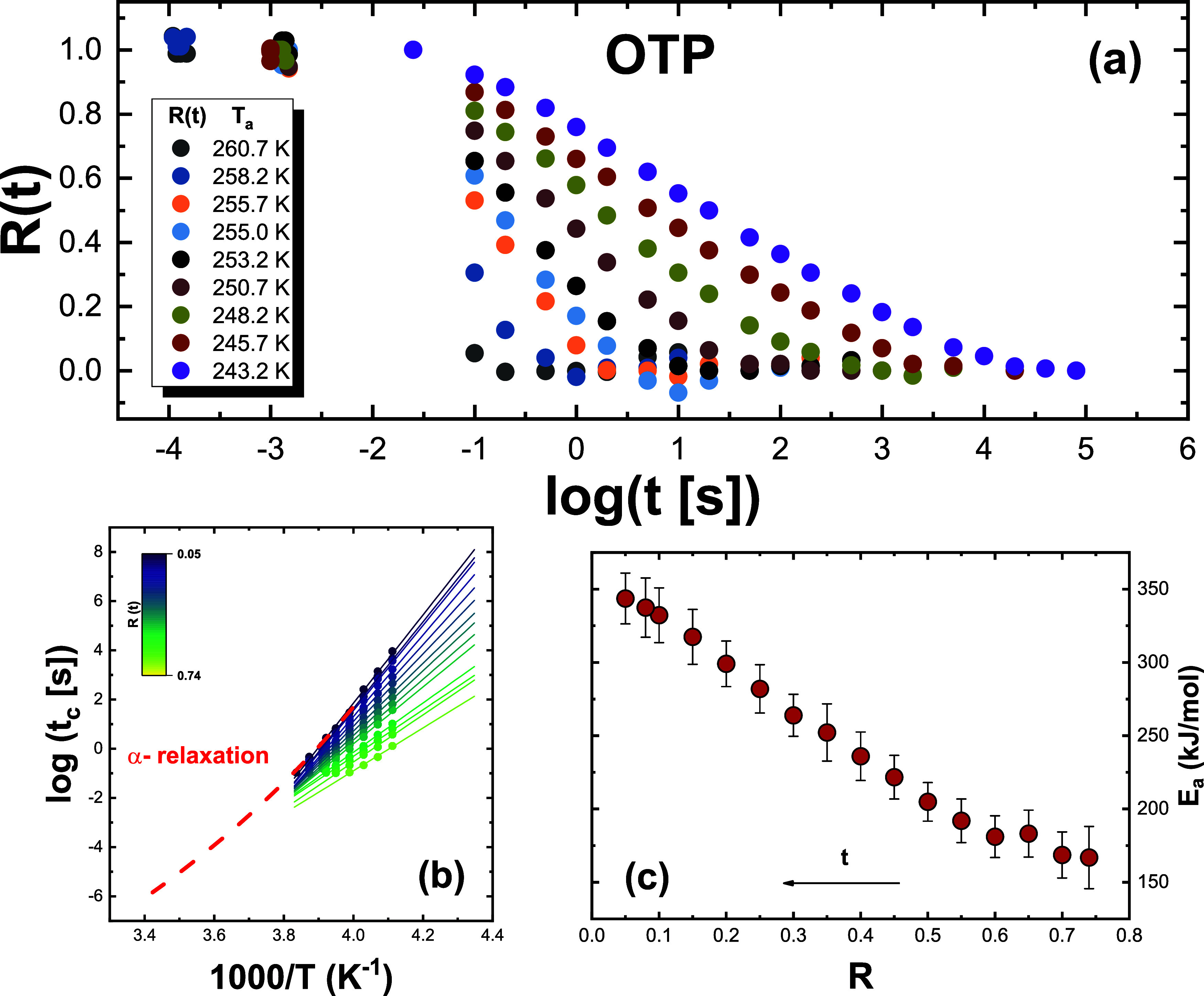
(a) Experimental data of the normalized relaxation function *R*(*t*) for all investigated aging temperatures for OTP (*T*_g_ = 263 at 1000 K s^–1^). (b) Logarithm of *t*_c_, the time to reach the degree of relaxation *R*(*t*), indicated in the color map as a function of the inverse temperature. The dashed line is the temperature dependence of the α-relaxation time taken from broadband dielectric spectroscopy (BDS) data,^45^ and it has been shifted by log *t* = +2 to match the experimental data. (c) Dependence of the activation energy obtained from the isoconversional method on the extent of aging.

**2:**
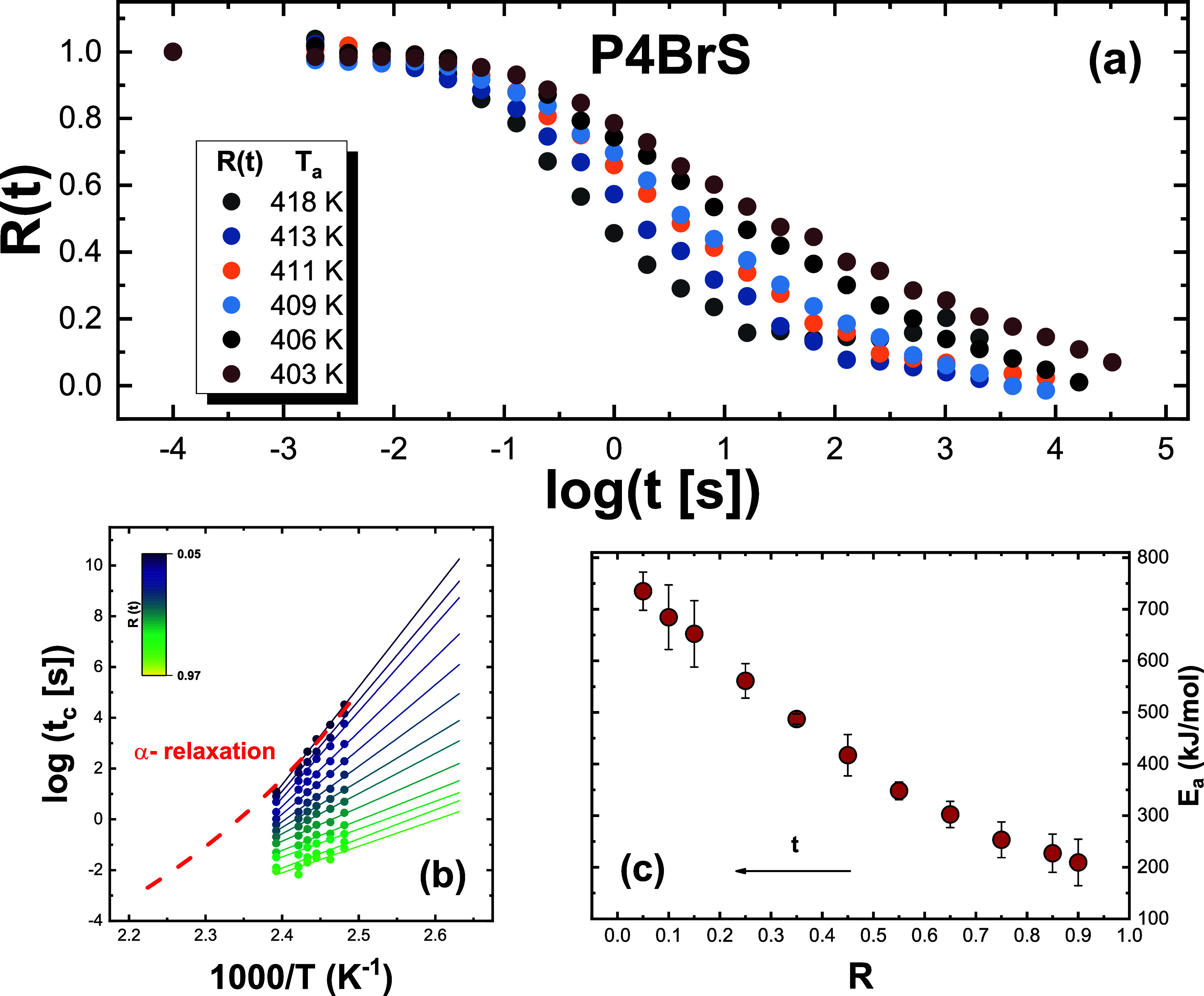
(a) Experimental data of the normalized relaxation function *R*(*t*) for all investigated aging temperatures for P4BrS (*T*_g_ = 435 at 1000 K s^–1^). (b) Logarithm of *t*_c_, the time to reach the degree of relaxation *R*(*t*), indicated in the color map as a function of the inverse temperature. The dashed line is the temperature dependence of the α-relaxation time taken from BDS data,^46^ and it has been shifted by log *t* = +0.65 to match the experimental data. (c) Dependence of the activation energy obtained from the isoconversional method on the extent of aging.

**Figure 3:**
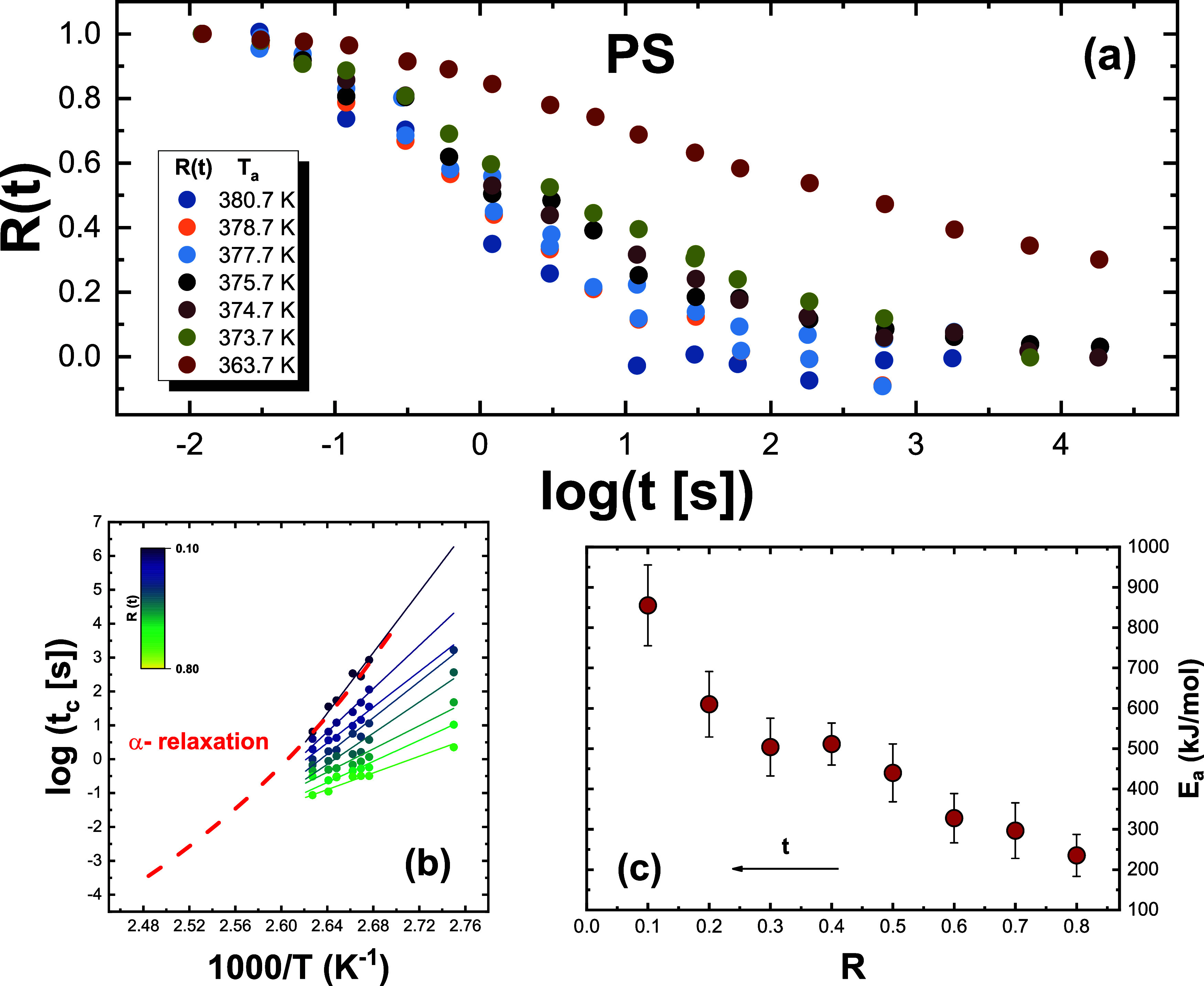
(a) Experimental data of the normalized relaxation function *R*(*t*) for all investigated aging temperatures for PS (*T*_g_ = 390 at 1000 K s^–1^). (b) Logarithm of *t*_c_, the time to reach the degree of relaxation *R*(*t*), indicated in the color map, as a function of the inverse temperature. The dashed line is the temperature dependence of the α-relaxation time taken from BDS data,^47^ and it has been shifted by log *t* = −0.22 to match the experimental data. (c) Dependence of the activation energy obtained from the isoconversional method on the extent of aging.

To perform isoconversional analysis, a certain degree of relaxation is needed. Since experimental data are typically taken at specific discrete values of the aging time, our data spectrum needs to be interpolated. This was performed in close proximity to the experimentally collected data. For each data set, we defined a discrete set of relaxation levels spaced at intervals of ≈0.05 and interpolated the time axis to determine the corresponding relaxation time scale, *t*_*R*_, the time required to reach a given extent of relaxation. Because the interpolation spacing is determined by *R*, this procedure results in a nearly logarithmic sampling of the time axis. For each temperature, interpolation was carried out within the experimentally accessible range of *R* values. Consequently, at higher temperatures—where only the later stages of the aging kinetics could be monitored—the data primarily contribute to lower relaxation levels. Other approaches, for example, interpolating *R* directly on a logarithmically spaced time axis and then determining *t*_*R*_, yield comparable outcome, confirming the robustness of the numerical implementation here employed in the isoconversional analysis. A graphical description on the way interpolation is performed in reported in Supporting Information (see Figure S2).

For experiments conducted under isothermal conditions, the activation energy, *E*_*R*_, at a given extent of relaxation *R*,is given by eq 1. Panels (b) of Figures 1–3 show the outcome of the analysis of relaxation data reported in panels (a) of the same figures for a discrete set of *R*(*t*) at different temperatures and times for OTP, P4BrS, and PS. Similar panels are shown in the Supporting Information for the other investigated glasses. Specifically, several isoconversional plots with log *t* vs 1000/*T* for different extents of *R*(*t*) are shown. As can be observed, as *R*(*t*) decreases, the slope of the linear fittings increases and, as a consequence, so does the effective activation energy during the aging process. This can be seen in panels (c) of Figures 1–3 for OTP, P4BrS, and PS (and the corresponding ones in the Supporting Information for the other glasses), where the dependence of the activation energy on the extent of relaxation is presented.

A common feature of the dependence of the activation energy on the extent of relaxation, in line with previous reports,^26,27^ is that it varies from a lower bound in the order of ∼100 kJ mol^–1^ to values as large as several hundreds kJ mol^–1^ at the end of the aging process. The latter can be compared to that of the main α- relaxation, whose temperature dependence of the typical relaxation time, conveniently shifted to match aging times, is presented in panels (b) of Figures 1–3 (and the corresponding panels in the Supporting Information for the other glasses). As can be observed, in all cases, the activation energy at the end of the aging process matches with that of the α-relaxation, indicating that the latter is the leading mechanism mediating the final stages of approach to equilibrium. Here, it is worth remarking that the shift of the α relaxation times is described within the Frenkel–Kobeko–Reiner (FKR) framework: β_c_ = *C*τ_α_^–1^, where *C* = Δlog *t* is the logarithmic shift, meaning the number of time decades, which needs to be added to log­(τ_α_) to match the logarithm of the cooling rate, log­(β_c_), which sets the vitrification temperature.^7^ In essence, the parameter *C* underlines the efficiency of the α relaxation in keeping the supercooled liquid at equilibrium on cooling.

The relatively low activation energy at small and intermediate extents of relaxation could also be attributed to the role of the α-relaxation in nonequilibrium conditions. A wealth of experiments carried out in the nonequilibrium glass actually show that the measured relaxation time exhibits moderate activation energy,^48–55^ in line with the outcome of our analysis. However, this interpretation would entail a discontinuity of the temperature dependence of the α-relaxation time as the system transforms from the supercooled liquid to the nonequilibrium glass. This scenario is at odds with the description of aging kinetics as a single activated event.^56^ Specifically, within the thermally activated description of the dynamics of glass forming systems, the freezing of configurational degrees of freedom leading to vitrification results in a temperature independent activation energy equal to that of the equilibrium systems just before vitrification. Seen from the viewpoint of the Adam–Gibbs theory,^57^ the aforementioned freezing implies that the configurational entropy remains constant below *T*_g_ and equal to that of the supercooled liquid before vitrification takes over.

Given these premises, the discontinuity in the glass activation energy with respect to that of the equilibrium supercooled liquid warrants an interpretation based on the role of other molecular mechanisms mediating physical aging at weak and moderate extents of relaxation. The most immediate candidate would be the β-relaxation detected by standard spectroscopic techniques.^58^ This was actually the interpretation in studies where isoconversional analysis conveyed a relaxation-dependent evolution of the activation energy analogous to that of our work.^26,27^ Furthermore, this is in line with recent analysis on a metallic glasses where the kinetics of devitrification a previously aged glass was shown to be sequentually mediated by γ, β, and ultimately α relaxation.^28^ However, among the investigated glass formers, KDE and BMMPC do not exhibit any trace of a β-relaxation by dielectric relaxation spectroscopy.^59,60^ Nevertheless, their behavior in terms of relaxation-dependent activation energy, as detected by isoconversional analysis, is completely analogous to that of their homologous glass formers, PDE and BMPC, where a secondary relaxation is clearly visible in dielectric relaxation spectroscopy experiments.^59,60^

The absence of β-relaxation in KDE and BMMPC may reflect limitations of the technique rather than a genuine lack of secondary dynamics. Thus, β-processes cannot be entirely ruled out. We also stress that recent work^61^ proposes that the β relaxation is best regarded as a generic process associated with local equilibration in the heterogeneous glassy structure, and activation energies extracted from calorimetric aging experiments often reflect mixed α–β dynamics rather than a single, species-specific mechanism. At the same time, the systematic observation of low-*E*_a_ values across different glass formers suggests the involvement of a more universal mechanism. A strong candidate is the slow Arrhenius process (SAP),^62^ a relaxation mode consistently identified in both polymers^63,64^ and small molecule glasses,^65,66^ and commonly ascribed to collective small displacements (CSD) involving localized, constrained rearrangements of molecular groups.^67^ Unlike the β-relaxation, which is often material-specific and sensitive to detection methods, SAP is characterized by a nearly temperature-invariant activation barrier, with values ranging from roughly 30–200 kJ mol^–1^ depending on the system. Importantly, the SAP has also been associated with other equilibration mechanisms in molecular glasses,^68–70^ further strengthening its relevance as a generic contributor to structural recovery both above and below *T*_g_.

Within this framework, the low thermal barriers detected as *R* → 1 could be attributed to SAP-mediated rearrangements, which remain active even deep below *T*_g_, where the α-process is essentially frozen. Previous studies have already correlated the first regime of physical aging kinetics with the SAP,^68^ associating the early stages of equilibration with these localized displacements, before the system gradually crosses over to the higher barriers characteristic of α-controlled recovery. Our isoconversional results reinforce this view: the activation energy initially reflects the SAP scale and then progressively increases, converging to α-relaxation at larger extents of relaxation. This supports a two-step equilibration scenario in which the SAP governs the onset of aging and the α-process dominates the final approach to equilibrium.

We remark that, in some cases, the present analysis depicts an even more complex scenario than that where a monotonic increase in the activation energy is observed. In particular, data obtained for PS^40^ and P4ClS,^41^ including isothermal aging experiments spanning a large temperature range down to temperatures significantly below *T*_g_, reveal that *E*(*R*) is no longer described by a smooth, monotonous function. Instead, distinct features emerge in the evolution of *E*(*R*), suggesting the occurrence of multiple consecutive steps in the aging process. Such complexity appears to be a general feature of glass-forming systems, as similar multistep aging dynamics have been reported in small organic molecules,^71^ polymers,^25,29,30,72,73^ metallic,^32,33^ and chalcogenide^31,74^ glasses.

In providing the value of the activation energy at given degrees of relaxation, isoconversional analysis can provide insights on the respective roles of β-relaxations, and the slow Arrhenius process (SAP), thereby improving our ability to distinguish between these mechanisms and to quantify their contributions to the early stages of physical aging. However, while isoconversional methods are very powerful because of their model-free character, their applicability is intrinsically limited to regions of the kinetics, where the extent of relaxation evolves measurably with time. As a consequence, they do not allow one to reliably access the limiting activation barriers in the asymptotic regimes *R* → 0 and *R* → 1, nor in the presence of intermediate plateaus associated with nearly invariant relaxation rates,^25^ owing to their mathematical definition. In such flat regions of the kinetics, the characteristic time associated with a given relaxation becomes poorly defined, and even small experimental noise in the relaxation can translate into large uncertainties in the extracted times and, consequently, in the apparent activation energies. Access to these limiting barriers therefore requires dedicated experiments spanning sufficiently broad time scales to explicitly include regimes where the kinetics becomes effectively time independent, as occurs at very short and very long times. A promising strategy to disentangle their roles lies in combining isoconversional and inflectional analyses. In such cases, the latter strategy, based on determining via model-free approaches the time scales associated with changes in the logarithmic slopes of *R*(*t*), as originally proposed by Kovacs,^75^ provides a viable route. Extending this approach to systems in which the activation barriers of the SAP and the β process are known and sufficiently distinct would enable quantitative separation of their respective contributions. In parallel, systematic aging studies performed far below *T*_g_ will be essential to capture the complex, nonmonotonic evolution of *E*(*R*) and to unravel the interplay of relaxation mechanisms governing long-term equilibration in glassy materials, where different processes are expected to become increasingly separated, potentially allowing distinct γ, β/SAP and α activation barriers to emerge also in organic glasses.

## Conclusions

In summary, by applying isoconversional analysis to a broad set of physical aging data on polymers and small molecule glasses, we have mapped the evolution of the effective activation barrier as a function of the extent of relaxation. Our results reveal a systematic increase of the activation energy from relatively small extent of relaxation to values comparable with the α-relaxation at large extents of relaxation. The low barriers detected at the onset of relaxation indicate that, in addition to the α-process, *additional molecular mechanisms must contribute to equilibration*. These early stages could involve secondary relaxations, such as the β-process, but the systematic character of the low activation energies across different systems points to the relevance of the slow Arrhenius process (SAP), associated with localized collective displacements and nearly temperature-invariant barriers. Taken together, our findings support a multiple-step scenario of glass equilibration in which low-barrier processes mediate the initial stages of aging, while the α-relaxation dominates the final approach to equilibrium. Disentangling the relative contributions of β-relaxations and the SAP is warranted in order to build a comprehensive picture of the microscopic dynamics underlying early stage glass aging.

## Supplementary Material



## Notes

Experimental procedures and results for the other glass formers not shown in the main manuscript (PDF)

## ∥

SISSA-Scuola Internazionale Superiore di Studi Avanzati, 34136 Trieste, Italy
