# Supplementary material for: Identifying the Thermal Barriers of Glass Aging via Isoconversional Analysis
Source: J Phys Chem B. 2026 Jan 26;130(5):1716–23. doi: 10.1021/acs.jpcb.5c07109 (PMC12884514; doi:10.1021/acs.jpcb.5c07109)
Supplement: Supplementary file 1 [file jp5c07109_si_001.pdf]

Identifying the Thermal Barriers of Glass Aging via Isoconversional Analysis

Vasiliki Maria Stavropoulou,[†,‡] , Federico Caporaletti,[§] Florian Pabst,[§||]
Valerio Di Lisio,[†,¶] Simone Napolitano, [§] and Daniele Cangialosi[*,#]

[†]Centro de Física de Materiales (CSIC–UPV/EHU), Paseo Manuel de Lardizábal 5, 20018 San Sebastián, Spain

[‡]PMAS, Faculty of Chemistry, University of the Basque Country (UPV/EHU), Paseo Manuel Lardizábal 3, 20018 Donostia-San Sebastián, Spain

[§]Laboratory of Polymer and Soft Matter Dynamics, Experimental Soft Matter and Thermal Physics (EST), Université libre de Bruxelles (ULB), Brussels, 1050, Belgium

[¶]Donostia International Physics Center, Paseo Manuel de Lardizábal 4, 20018 San Sebastián, Spain

[||]Current address: SISSA-Scuola Internazionale Superiore di Studi Avanzati, 34136 Trieste, Italy

Materials

Poly(4-bromostyrene) (P4BrS) with molecular weight of M_w=6.5 ×10^4 g/mol was purchased from Sigma Aldrich and used as received.

Fast scanning calorimetry measurements

Fast Scanning Calorimetry (FSC) measurements were performed with a Mettler-Toledo Flash Differential Scanning Calorimeter (DSC 2+) equipped with an intracooling stage. The sample chamber was maintained under a dry nitrogen atmosphere (20 ml/min). The investigated materials were directly deposited onto Mettler-Toledo UFS 1 chips.

The thermal protocol for aging experiments is illustrated in Fig. S1. Samples were first heated to ~T_g + 100K, then quenched at a cooling rate of q = 1000 Ks^{-1} to the aging temperature T_a, where they were held isothermally for a variable time t_a. After each isothermal hold, samples were quenched to 183 K at 1000 Ks^{-1}, followed by the heating scan for data collection with a heating rate of 1000 Ks^{-1}.

Physical aging was identified by the development of an endothermic overshoot upon heating, superimposed on the specific-heat step at the glass transition (see Fig. S1). The

overshoot grows with increasing t_a and shifts to higher temperatures as the glass approaches more stable thermodynamic states. The degree of equilibration was quantified via the fictive temperature $T_f\,(t_a, T_a)$, evaluated using Moynihan's area matching method,[1] following standard practice in the field.

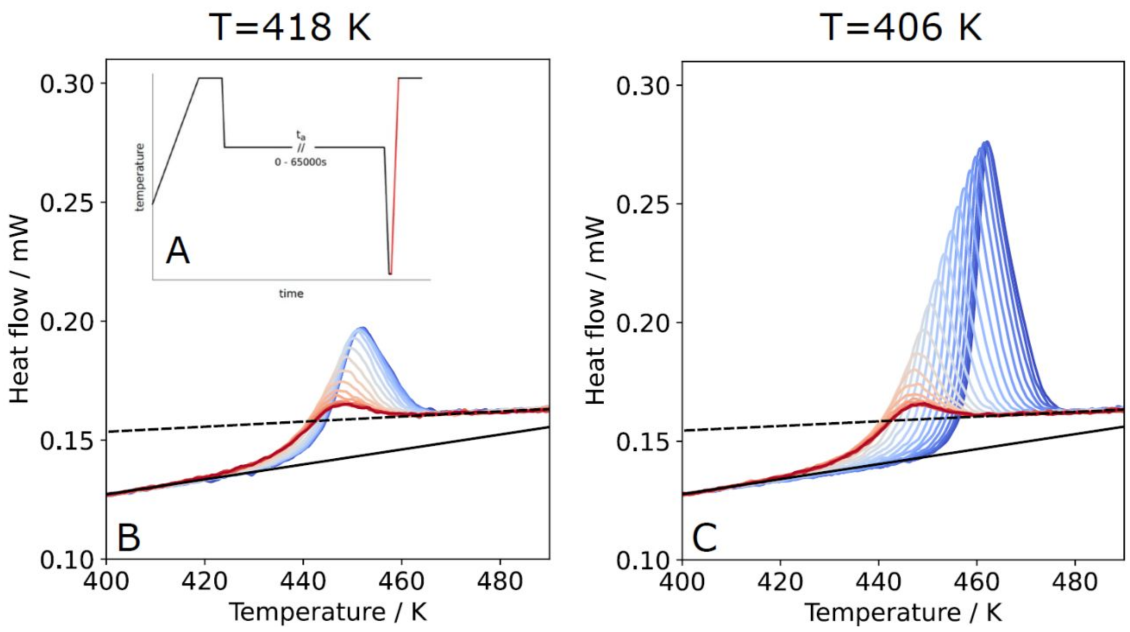

Figure S1: *FSC scans of P4BrS after aging between 0.01 and 32768 s with a sampling power with base 2 at the indicated temperatures. The inset shows the thermal protocol for FSC experiments.*

Interpolating the relaxation function

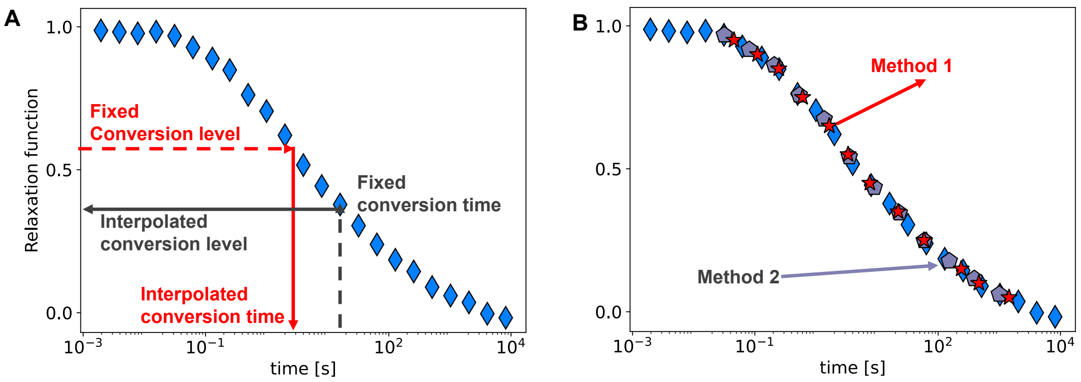

Figure S2. *Example of the interpolation protocols used to process the relaxation functions analyzed in this work. Blue diamonds in panels A and B represent experimental data from a P4BrS sample quenched at 1000 K/s from the liquid state to an aging temperature of 409 K.*

Panel A: Two interpolation procedures are illustrated. Method 1 (red): fixed conversion levels are selected along the relaxation function, and the corresponding times are obtained by interpolating the time axis. Method 2 (gray): the relaxation function is interpolated on a logarithmically spaced time grid.

Panel B: Comparison of the two methods, which provides nearly identical interpolated functions. In this work, Method 1 was adopted, as it facilitates the selection of conversion levels close to the experimental data, thereby minimizing potential bias. Nevertheless, the similarity between the two interpolations confirms the robustness of the isoconversional analysis, independently of the interpolation protocol.

Supplementary figures on isoconversional analysis

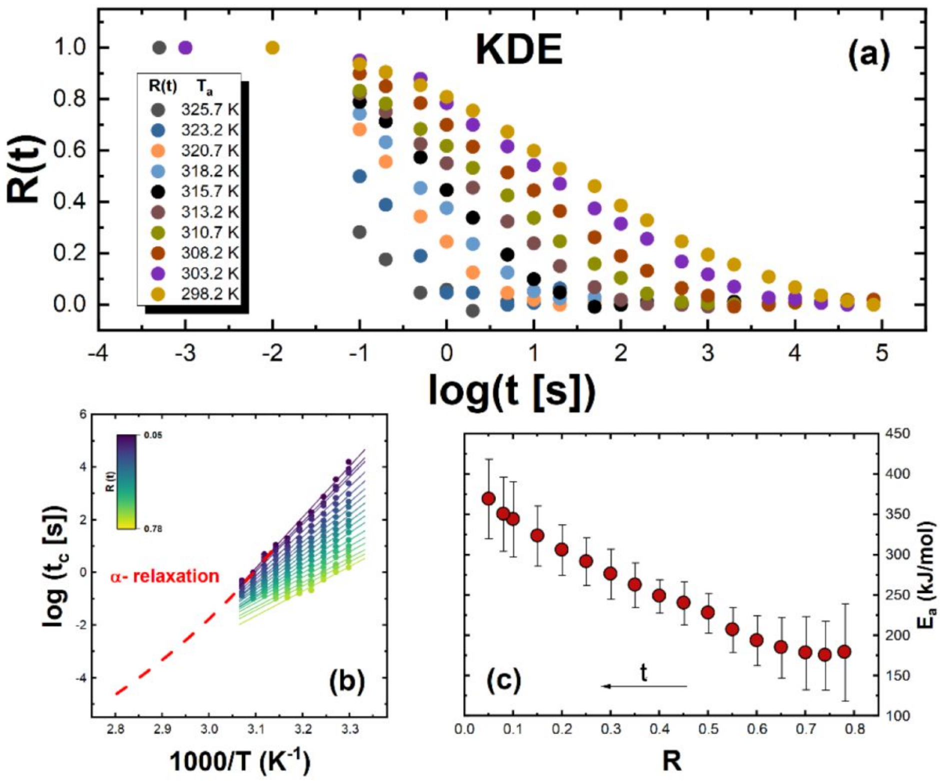

Figure S3: *(a) Experimental data for o-cresolphthalein dimethyl ether (KDE) (T_g = 330.5 K at 1000 Ks^{-1}) of the normalized relaxation function R(t) vs aging time for all investigated aging temperatures. (b) Time to reach the indicated degree of relaxation as a function R(t) of the inverse temperature. The dashed line is the temperature dependence of the α relaxation time taken from ref. [1] for BDS and it has been shifted by $log\, t = +0.35$ to match the experimental data. (c) Dependence of the activation energy obtained from the isoconversional method on the extent of aging.*

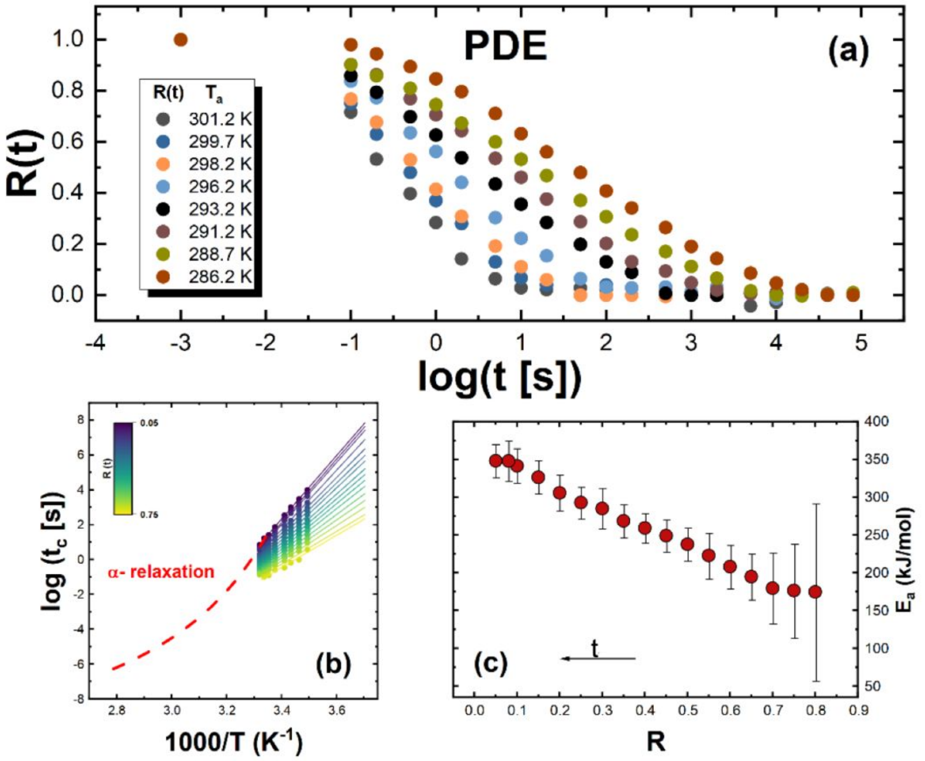

Figure S4: Same as in Figure S3 for phenolphthalein dimethyl ether (PDE) (T_g = 306.5 K at 1000 Ks^{-1}). The temperature dependence of the α relaxation time is taken from Ref. [2] and it has been shifted by $\log t = +0.65$ to match the experimental data.

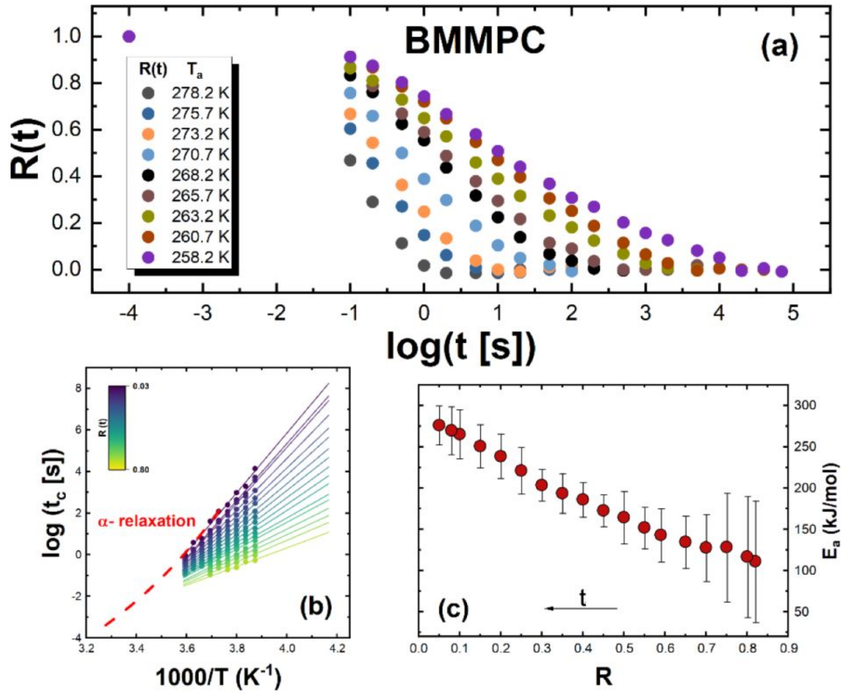

Figure S5: *Same as in Figure S3 for 1,1-bis (4-methoxyphenyl)cyclohexane (BMMPC) (T_g = 277 K at 1000 Ks^{-1}). The temperature dependence of the α relaxation time is taken from Ref. [2] and it has been shifted by* $\log t = +1.70$ *to match the experimental data.*

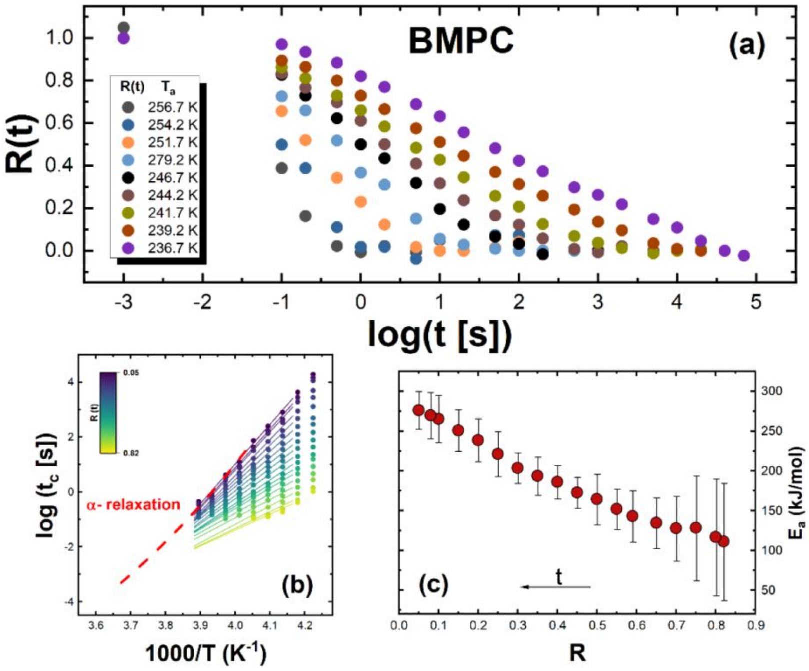

Figure S6: *Same as in Figure S3 for 1,1-di(pmethoxyphenyl)cyclohexane (BMPC) (T_g = 259 K at 1000 Ks^{-1}). The temperature dependence of the α relaxation time taken from Ref. [2] and it has been shifted by* $\log t = +1.50$ *to match the experimental data.*

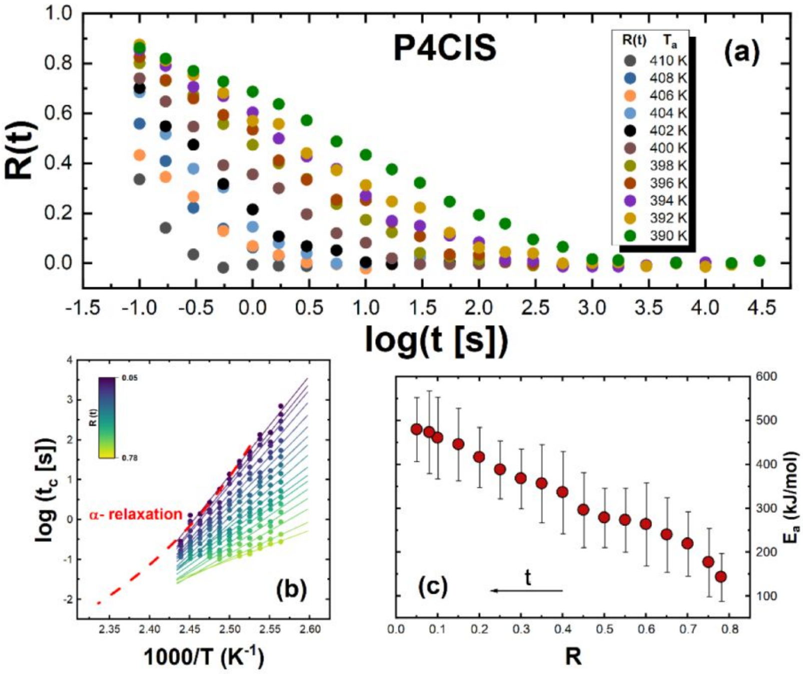

Figure S7: *Same as in Figure S3 for poly(4-chloro styrene) (P4ClS). The temperature dependence of the α relaxation time taken from Ref.* [3,4] *and it has been shifted by* $\log t = +2.70$ *to match the experimental data.*

References

1. Kahle S, Gapinski ; J, Hinze ; G *et al.* A comparison of relaxation processes in structurally related van der Waals glass formers: The role of internal degrees of freedom. J Chem Phys 2005; 122: 59.

2. Dreyfus C, Le Grand A, Gapinski J, Steffen W, Patkowski A. Scaling the α-relaxation time of supercooled fragile organic liquids. The European Physical Journal B - Condensed Matter and Complex Systems 2004; 42: 309–319.

3. Di Lisio V, Rocchi LA, Cangialosi D. Twofold Facet of Kinetics of Glass Aging. Phys Rev Lett 2024; 133: 048201.

4. Song Z, Rodríguez-Tinoco C, Mathew A, Napolitano S. Fast equilibration mechanisms in disordered materials mediated by slow liquid dynamics. Sci Adv 2022; 8: 7154.
